# Adverse events in patients treated with neoadjuvant chemo/immunotherapy for triple negative breast cancer: results from seven academic medical centers

**DOI:** 10.1007/s10549-025-07758-8

**Published:** 2025-07-04

**Authors:** Jessica Mezzanotte-Sharpe, Chih-Yuan Hsu, David Choi, Hollie Sheffield, Sara Zelinskas, Ekaterina Proskuriakova, Mateo Montalvo, Danelle S. Lee, Jennifer G. Whisenant, Keaton Gaffney, Michael S. Thompson, Kim Blenman, Karine Tawagi, Lynn Symonds, Cesar Santa-Maria, Nisha Unni, Dionisia Quiroga, Yu Shyr, Laura C. Kennedy

**Affiliations:** 1 Division of Hematology and Oncology, Vanderbilt University Medical Center, 2200 Pierce Avenue, 777 PRB, Nashville, TN 37232-6307 USA; 2 Department of Biostatistics, Vanderbilt University Medical Center, Nashville, TN USA; 3 Johns Hopkins Sidney Kimmel Comprehensive Cancer Center, Baltimore, MD USA; 4 Division of Hematology/Oncology, University of Texas Southwestern Medical Center, Dallas, TX USA; 5 Division of Medical Oncology, The Ohio State University Comprehensive Cancer Center, Columbus, OH USA; 6 Department of Medicine, University of Illinois Chicago, Chicago, IL USA; 7 Sinai Chicago Internal Medicine, Chicago, IL USA; 8 Section of Medical Oncology, Yale School of Medicine, New Haven, CT USA; 9 Clinical Pharmacy, Vanderbilt University Medical Center, Nashville, TN USA; 10 Division of Hematology/Oncology, University of Illinois Chicago, Chicago, IL USA; 11 Department of Medicine, University of Washington School of Medicine, Seattle, WA USA; 12 Clinical Research Division, Fred Hutchinson Cancer Center, Seattle, WA USA

**Keywords:** Triple negative breast cancer, Immune-related adverse events, Pembrolizumab

## Abstract

**Purpose:**

The standard-of-care neoadjuvant treatment for early-stage or locally advanced triple negative breast cancer (TNBC) is the KEYNOTE-522 regimen that combines pembrolizumab and chemotherapy. Although this approach has superior response and survival rates, high-grade adverse events (AEs) are common. Real-world data from a diverse patient population is needed to better understand practice patterns and the impact of immunotherapy in TNBC patients.

**Methods:**

Medical records from TNBC patients were retrospectively reviewed during neoadjuvant and adjuvant treatment with pembrolizumab and chemotherapy. CTCAE version 5.0 was used to grade AEs. Variables were reported with descriptive statistics, and AE, pCR and hospitalization rates were estimated with 95% confidence intervals.

**Results:**

We identified 415 patients from seven academic medical centers; 60% identified as White and 21% as Black. pCR rate was 52%. 88% of patients experienced an AE, 38% experienced a grade 3+ AE, and 31% stopped pembrolizumab early. Hospitalization rate was 26%. There were no statistically significant differences in AE, pCR or hospitalization rates between White and Black patients. Obese patients had a statistically significant higher hospitalization rate (p = 0.014). There were 18 deaths during treatment, mainly from progressive TNBC.

**Conclusion:**

This is one of the largest real-world, diverse patient cohorts for TNBC patients treated with chemotherapy and pembrolizumab. pCR rate was lower than that reported in the KEYNOTE-522 study and in smaller real-world studies, potentially due to high rates of pembrolizumab and chemotherapy discontinuation. AEs and hospitalizations were common, with obese patients more likely to be hospitalized than patients with a normal BMI.

**Supplementary Information:**

The online version contains supplementary material available at 10.1007/s10549-025-07758-8.

## Introduction

Approximately 15% of newly diagnosed breast cancers are triple negative [1]. Triple negative breast cancer (TNBC) is defined by the lack of expression of hormone receptors or lack of overexpression of the human epidermal growth factor receptor 2 (HER2), and as such, TNBC traditionally derives limited benefit from targeted therapies. TNBC has higher risks of distant recurrence and death than hormone receptor-positive disease and is therefore treated aggressively in the curative-intent setting [1]. Historically, the standard-of-care treatment for early-stage or locally advanced TNBC consisted of multi-agent chemotherapy given in the neoadjuvant setting, as early studies showed that assessing tumor response at the time of surgery led to the ability to prognosticate based on tumor response to treatment [2]. Patients achieving a pathologic complete response (pCR) at the time of surgery have an estimated relapse-free 10-year survival rate of greater than 80% [3]. In 2020, the results of the KEYNOTE-522 study examining the combination of neoadjuvant multi-agent chemotherapy with paclitaxel, carboplatin, doxorubicin/epirubicin and cyclophosphamide with the immune checkpoint inhibitor (ICI) pembrolizumab administered in both the neoadjuvant and adjuvant setting were published [4]. This regimen demonstrated a superior pCR rate compared to chemotherapy alone and improved disease free survival (DFS) and overall survival (OS) [4–6], making this the new standard-of-care treatment strategy for early-stage or locally advanced TNBC.

Combining immunotherapy with chemotherapy is not without risks. In addition to the known side effects from chemotherapy, immune-related adverse events (irAEs) secondary to ICI treatment have been extensively described [7–9]. The KEYNOTE-522 study reported an overall adverse event (AE) rate of 99.2% in the pembrolizumab-chemotherapy treatment arm and a 33.5% rate of any grade irAE [5]. Of the irAEs, 12.9% were grade 3 or higher, and one patient in the pembrolizumab-chemotherapy group died of an irAE (pneumonitis) [5]. The average age of patients in the KEYNOTE-522 study population was 49 years old, and race was not reported [4]. Subsequently, there have been several real-world studies examining irAE rates in patients undergoing treatment with this regimen [10–17]. These have mostly shown higher rates of irAEs than the KEYNOTE-522 study but have shown similar rates of pCR [10, 11, 13, 15]. Only three studies included data from more than 100 patients (and the largest sample size was 233 patients) who received chemotherapy and pembrolizumab [10, 13, 14].

The goal of our study was to therefore examine treatment practices and AEs in a large, racially diverse cohort of TNBC patients undergoing neoadjuvant treatment with chemotherapy and neoadjuvant and adjuvant pembrolizumab. Additionally, since obese patients are more likely to experience AEs from chemotherapy and immunotherapy than patients of normal weight (reviewed in [18]), we explored the impact of patient BMI on pCR and AEs. We enrolled seven academic medical centers and collected data on 415 eligible patients. We found that patients’ chemotherapy and/or pembrolizumab were frequently discontinued due to AEs and that hospitalization during treatment was common, particularly in obese patients. Additionally, the pCR rate for our patient cohort was one of the lowest reported in a real-world study at 52%. This is one of the largest studies showing real-world practice data for TNBC patients undergoing neoadjuvant chemotherapy and neoadjuvant and adjuvant pembrolizumab treatment.

## Methods

### Patient identification and data collection

Seven academic medical centers participated in this study. Adult patients (defined as age 18 or older) with TNBC as assessed by their treating provider who were prescribed neoadjuvant pembrolizumab and chemotherapy between 2021 and 2024 were retrospectively identified through pharmacy treatment records. Only patients who had completed neoadjuvant treatment and had a surgical pathology result available were eligible for this study. Patients did not have to start or complete adjuvant treatment, although we did collect treatment information for patients who had started adjuvant therapy.

Electronic medical records, including laboratory values and treating provider notes, were reviewed for data collection. Study data were collected and managed using REDCap electronic data capture tools hosted at Vanderbilt University Medical Center [19, 20]. Research Electronic Data Capture (REDCap) is a secure, web-based software platform designed to support data capture for research studies, providing (1) an intuitive interface for validated data capture; (2) audit trails for tracking data manipulation and export procedures; (3) automated export procedures for seamless data downloads to common statistical packages; and (4) procedures for data integration and interoperability with external sources.

We reviewed demographic, clinicopathologic, and treatment-related data. Variables collected included the following: age at breast cancer diagnosis, race, ethnicity, sex, baseline ECOG PS, baseline BMI, death and whether death was related to breast cancer or treatment-related AEs, tumor stage (AJCC 8th edition clinical prognostic staging system), breast surgery type, timing of breast surgery, chemotherapy regimen and number of doses of each agent received, pembrolizumab doses received in the neoadjuvant and adjuvant settings, use of growth factor support, pCR status, residual cancer burden status, number of hospitalizations and admitting diagnoses/other diagnoses of note, and treatment-related AEs/irAEs including type and grade of AE. The Common Terminology Criteria for Adverse Events (CTCAE) version 5.0 was used to grade toxicities. When a toxicity event was unable to be assessed, it was not assigned a grade and was not listed as a grade 3+ AE.

### Statistical analysis

Descriptive statistics were used to report all variables. Total adverse event (AE) rates, grade 3+ AE rates, pCR rates and hospitalization rates for patient groups were estimated, where the 95% confidence intervals were calculated by 5000 Monte Carlo simulations. Event rates between groups were compared using Fisher’s exact test. A p-value of < 0.05 indicates the rate in one group is significantly different from those in other groups. The difference in proportions between any two groups was evaluated using two sample proportions test, where the 95% confidence intervals and p-values were calculated by 5000 Monte Carlo simulations. All analyses were performed with R software version 4.3.1 (R Foundation for Statistical Computing).

### Ethics statement

This project was approved by the Vanderbilt University Medical Center Institutional Review Board (IRB #231200, date of approval 7/27/2023), and a waiver of informed consent was granted by the VUMC IRB. Each participating site obtained IRB approval: University of Texas Southwestern Medical Center, The Ohio State University Wexner Medical Center, Johns Hopkins University, University of Illinois Chicago, University of Washington and Yale University.

## Results

### Patient demographics

Patient demographics are shown in Table 1. Briefly, 415 patients were eligible. Median age at treatment initiation was 50 years old, with an age range of 22 to 84 years old. All patients were female, and 17% were age 65 or older. Sixty percent identified as White, and 21% percent identified as Black. Seven percent identified as Hispanic or Latino. Tumor stage information using the AJCC 8th edition clinical prognostic staging system is provided in Table 1. Almost all patients (94%) had stage II or III disease.

**Table 1:**
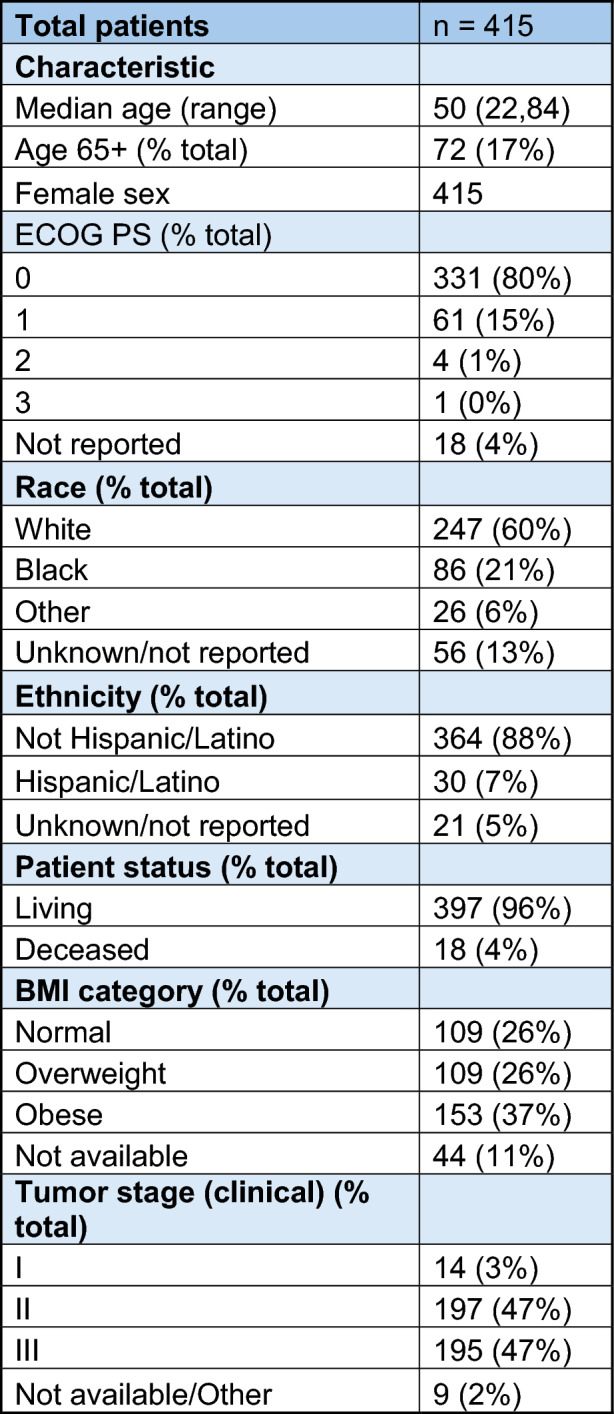
Patient demographics

*ECOG PS* Eastern Cooperative Oncology Group Performance Status

Patients were categorized by BMI into normal (BMI range 18.5–25 kg/m^2^), overweight (BMI 25–less than 30 kg/m^2^), or obese (BMI 30 kg/m^2^ or greater) based on baseline BMI. Most patients (63%) were overweight or obese.

### Treatment information

Patients were eligible for this study if they underwent treatment with any combination of chemotherapy and pembrolizumab for curative-intent for TNBC. Clinical trial patients were included. Most patients in this analysis underwent treatment with the KEYNOTE-522 regimen. One patient underwent treatment with a regimen that was not included in the dosing analysis, and two other patients were not included in the dosing analysis for carboplatin or paclitaxel because they were on other regimens before being treated with AC and pembrolizumab. The number of doses of chemotherapy agents and pembrolizumab are shown in Table 2. Information on dose reductions and/or treatment delays was not collected. For patients receiving weekly carboplatin, 65% completed treatment with 12 or more doses. For patients receiving carboplatin every 3 weeks, 92% completed treatment with four or more doses. 79% of patients received full treatment with paclitaxel (12 or more doses), and 78% received full treatment (4 or more doses) of doxorubicin/epirubicin and cyclophosphamide (AC/EC). Of note, some sites used dose-dense AC, while some patients received standard dose AC. Details on patients who received dose dense versus standard dosing were not recorded. The majority of patients (76%) also received growth factor support at some point during treatment.

**Table 2:**
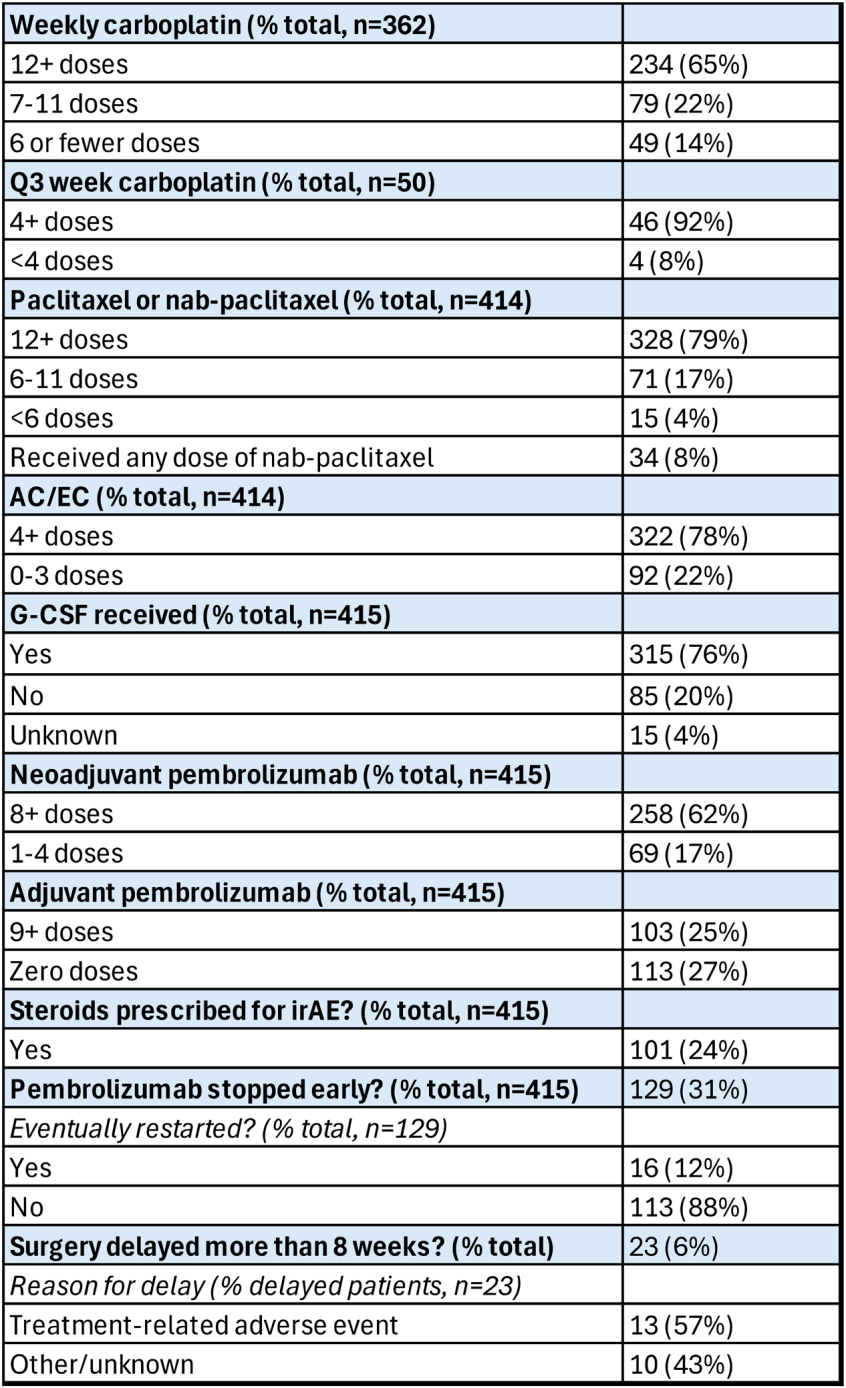
Treatment information

*Q3* every 3, *AC/EC* adriamycin/cyclophosphamide or epirubicin/cyclophosphamide, *irAE* immune-related adverse event

For pembrolizumab, 62% of patients completed neoadjuvant treatment with 8 or more doses (Table 2). For neoadjuvant pembrolizumab, five patients received one or fewer doses (although one patient who received no neoadjuvant pembrolizumab did go on to complete adjuvant therapy with pembrolizumab); 69 patients (17% of the total) received 4 or fewer doses, and 59 patients (14% of the total) received 6 or 7 doses. In adjuvant setting, 25% (103 out of 415) of patients completed pembrolizumab with 9 or more doses, and 39% (162 out of 415) completed 8 or more doses. A significant portion of patients (113 out of 415, or 27%) did not receive any doses of adjuvant pembrolizumab. Of these patients, 67 (16%) did not receive adjuvant pembrolizumab due to a documented adverse event, 4 (1%) did not receive adjuvant pembrolizumab due to progression of disease, and 33 (8%) did not receive adjuvant pembrolizumab due to other reasons, the majority of which were documented as patients declining to pursue adjuvant therapy. Only 9 patients in our study population (2%) had not received a dose of adjuvant pembrolizumab because they had just completed surgery and were waiting to start adjuvant treatment. Because every patient in this study had not completed their adjuvant therapy at the time of data collection, the study asked whether pembrolizumab was stopped early. 31% of patients had pembrolizumab stopped early, and it was rarely re-started once stopped.

We also examined surgery information and delays in surgery, which was defined as a surgery that took place more than 8 weeks after completing neoadjuvant treatment. Only 6% of patients experienced delayed surgeries, and half of the delays were due to documented treatment-related AEs. Surgical information is displayed in Supplemental Table 1. Most patients underwent bilateral or unilateral mastectomy (65%), and 28% underwent axillary lymph node dissection compared to 70% who underwent a sentinel lymph node biopsy.

### Response rates

Surgical pathology results were available for every patient in this study. 52% achieved a pCR, while 48% had residual disease (Table 3). Of those with residual disease, residual cancer burden (RCB) scores were recorded when those were available. Results for RCB scores are shown in Table 3.

**Table 3:**
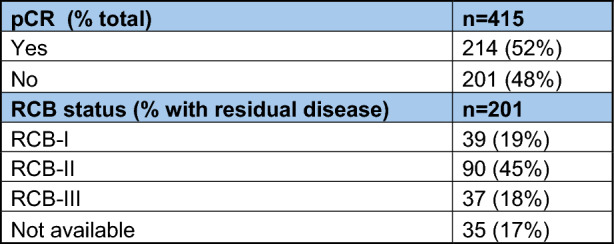
Response rates

*pCR* pathologic complete response, *RCB* residual cancer burden

### Adverse events (AEs) and hospitalizations

Adverse event information is reported in Table 4. In this study, 88% of patients experienced an AE, and 38% experienced a grade 3 or greater AE. The most common AE was myelosuppression, which included anemia, thrombocytopenia, or neutropenia. This also was the most common grade 3+ AE (28% of all patients). Other common side effects related to chemotherapy were reported, including nausea/vomiting, fatigue, mucositis, and electrolyte abnormalities.

**Table 4:**
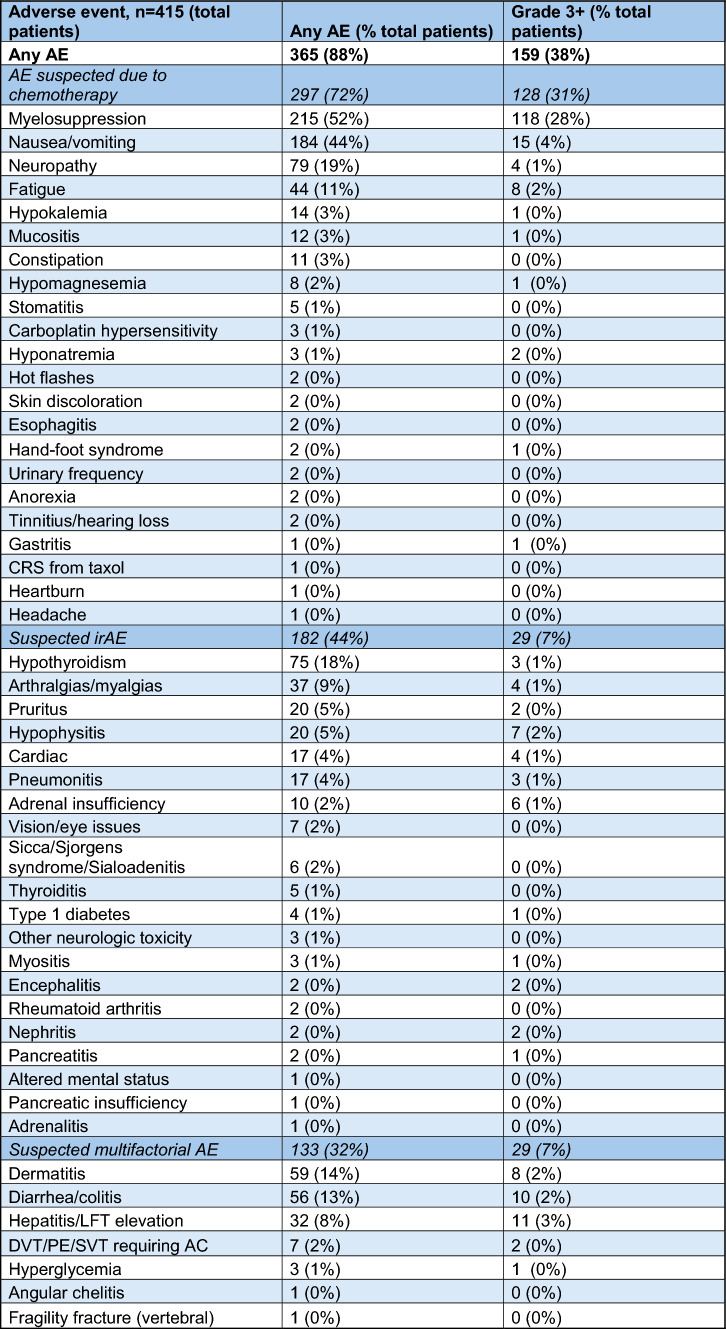
Adverse events

*AE* adverse event, *irAE* immune-related adverse event, *LFT* liver function test, *DVT* deep vein thrombosis, *PE* pulmonary embolism, *SVT* superficial venous thrombosis

The retrospective nature of this study made it difficult to determine which AEs were specifically related to pembrolizumab versus chemotherapy, so we have reported rates of suspected irAEs as well as AEs that were likely due to both immunotherapy and chemotherapy (referred to as multifactorial AEs in Table 4). Of these, hypothyroidism was the most common (18%), and dermatitis, pruritis, arthralgias/myalgias not defined as myositis, and diarrhea or colitis were also common. Although rare, there were several grade 3+ cases of adrenal insufficiency, and there were four grade 3+ cardiac irAEs (two cases of myositis and two cases of arterial thrombosis). Pneumonitis was also reported, although only 3 of 17 cases were grade 3+. There were also rare irAEs, including the development of type 1 diabetes, rheumatoid arthritis, and encephalitis. Interestingly, nephritis was rare, with only 2 cases reported (both grade 3+). Out of the 253 patients who experienced a suspected irAE or multifactorial AE (61% of the total population), the pCR rate was similar to the overall population at 54.5%. For patients with grade 3+ irAEs or grade 3+ multifactorial AEs (51 patients total), the pCR rate was also similar at 52.9%.

This regimen was associated with a hospitalization rate of 26% (Table 5). This included inpatient admission or observation admission and did not include urgent care or emergency department visits. Most hospitalizations occurred in the neoadjuvant setting, and many patients experienced more than one hospitalization. Of the total number of hospitalizations, 39% had a suspected irAE documented during admission. The most common admitting diagnosis was neutropenic fever (46 out of 153 [30%] total hospitalizations), and the second most common admitting diagnosis was a suspected irAE (24% of the total hospitalizations). Of note, of the patients admitted with neutropenic fever, 80% had documentation of receiving some form of growth factor support during their neoadjuvant treatment, although since we did not collect information on the timing of G-CSF administration, most of these patients likely received G-CSF after their admission for neutropenic fever.

**Table 5:**
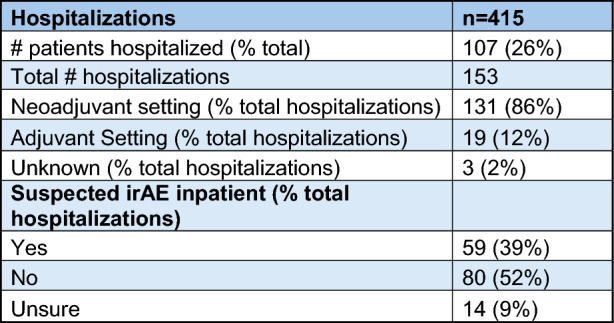
Hospitalization information

*irAE* immune-related adverse event

### Differences between groups

AE rates and grade 3+ AE rates were compared based on patient demographic factors. There were no differences in total AE rates between White and Black patients (89.9% in White patients compared to 82.6% in Black patients, estimated difference − 0.073, 95% confidence interval [− 0.166 to 0.013], p-value 0.098). Grade 3+ AE rates were also not significantly different although did trend towards a higher rate in White patients, with a 44.5% grade 3+ AE rate versus 32.6% in Black patients (estimated difference − 0.120, 95% CI [− 0.234 to 0.002], p = 0.053). There were also no differences in total or grade 3+ AE rates based on BMI category (normal weight, overweight, and obese). Patients younger than 65 and ages 65 and older did not have a difference in total AE rates (88.3% in younger patients compared to 86.1% in patients 65 and older, estimated difference − 0.022, 95% CI [− 0.114 to 0.060], p-value 0.647) or in grade 3+ AE rates.

pCR rates were also compared based on patient demographics. There were no differences in pCR rate between White and Black patients (49% in White patients and 52% in Black patients; estimated difference 0.033, 95% CI [− 0.95 to 0.156], p-value 0.605) or between any of the BMI categories. There was also not a difference in pCR rate between patients ages 65 and older or under age 65 (47% versus 52.5%, respectively; estimated difference − 0.053, 95% CI [− 0.180 to 0.078], p = 0.437). Grade 3+ AE rates were also compared between patients who did and did not experience a pCR and were not significantly different (41.8% grade 3+ AE rate in patients who did not experience a pCR compared to 35% in patients who had a pCR, estimated difference − 0.067, 95% CI [− 0.161 to 0.024], p = 0.150). Finally, patients who experienced a suspected irAE or suspected multifactorial AE did not have a statistically significant difference in pCR rate (54.5% in patients who experienced a suspected irAE versus 46.9% in patients without an irAE, estimated difference 0.076, 95% CI [− 0.021 to 0.172], p = 0.116).

We also examined differences in suspected irAEs or suspected multifactorial AEs (253 patients, or 61% of our study population) between groups. There were no statistically significant differences in irAE rate between White and Black patients (64.4% versus 55.8%, p = 0.169) or based on BMI category (56.9% irAE rate in normal weight, 61.5% in overweight, and 63.4% in obese patients). Although there was a 6.5% difference in irAE rate between normal weight versus obese patients, that result was not statistically significant (p = 0.294). There was also not a statistically significant difference in irAE rates between patients 65 and older verses those younger than 65 (55.6% versus 62.1%, p = 0.306) or when comparing patients under 50 to those ages 50 and older (61.5% versus 60.5%, p = 0.833).

Hospitalization rates (using first hospitalization only) were also examined. There were no differences in hospitalization rates between White and Black patients (29.1% versus 24.4%, p = 0.393) or between patients ages 65 and older or under 65 (25% versus 25.7%, p = 0.894). When comparing BMI categories, however, we found a statistically significant increase in hospitalizations for obese patients when compared to patients of normal weight (32% versus 18.5%, estimated difference 0.135, 95% CI [0.027 to 0.239], p-value 0.014). Overweight patients had a 27.5% hospitalization rate, although this was not statistically significant compared to normal weight or obese patients (p = 0.125 and p = 0.418, respectively).

## Discussion

This is one of the largest real-world studies examining treatment patterns and AEs in a diverse patient cohort for TNBC patients receiving neoadjuvant combination chemotherapy and pembrolizumab. pCR rates from available real-world studies range from 57.1 to 64.7% compared to 64.8% in the KEYNOTE-522 study [4, 10, 11, 13, 15, 17]. Our study’s pCR rate was lower than these at 52% and was more consistent with smaller studies reported by Wood et al. and McFarlane et al., who reported pCR rates of and 48.4% (n = 64 patients who had undergone surgery) and 45.5% (n = 23), respectively [12, 16]. While it is impossible to determine the exact cause of the lower pCR rate in our study given its retrospective nature, and while cross study comparison should be treated with caution, it is reassuring that pCR rates in our study were not significantly different between White and Black patients, overweight or obese patients compared to those of a normal weight, or younger and older patients. Grade 3+ AE rates were not different between patients who achieved or did not achieve a pCR, and rates of pCR in patients with any grade irAE/multifactorial AE or grade 3+ irAE/multifactorial irAE were also similar to the overall study population (54.5% and 52.9%, respectively).

One factor likely contributing to the lower rates of pCR seen in this patient cohort is the high rates of chemotherapy and pembrolizumab discontinuation. Notably, 19 patients (5%) in our study did not receive a single dose of carboplatin, and 22% (92 out of 414) did not receive all four doses of AC/EC. The KEYNOTE-522 study defined full chemotherapy exposure as carboplatin weekly 10–12 doses or four doses using every 3 week dosing, paclitaxel weekly 10–12 doses, and four doses of doxorubicin/epirubicin and cyclophosphamide [4]. Only 63% of the patients who were given the KEYNOTE-522 regimen in our study completed chemotherapy using those parameters. Some of this variation is likely due to changing practice patterns at the time the KEYNOTE-522 regimen was approved by the FDA, but much of the difficulty associated with completing this regimen is also due to the high rates of AEs and irAEs. With 31% of patients stopping pembrolizumab early during the course of their treatment (and often not receiving adjuvant therapy), ongoing studies are needed to assess rates of both response and recurrence as well as overall survival in this patient population. Studies like the ongoing phase III SCARLET trial (NCT05929768), which is examining using pembrolizumab with a taxane-platinum chemotherapy backbone and omits the anthracycline component of KEYNOTE-522, will be important to assess outcomes for patients who receive less total treatment but may be able to better complete all of their planned therapy if they experience fewer AEs.

Although we did not collect information on dose reductions or treatment delays, limiting some of the analysis regarding dosing information in this study, it is also interesting that more patients were able to complete all planned doses of their carboplatin chemotherapy if they received carboplatin every 3 weeks compared to weekly (92% versus 65%). A study of 153 TNBC patients showed that dose reductions were associated with lower pCR rates [10]. Specifically, patients receiving reduced doses of carboplatin (n = 46) had a pCR rate of 50% compared to those receiving full dose carboplatin (n = 103) at 73% (p = 0.01) [10]. Thus, the number of doses in addition to strength of doses plays an important role in determining a patient’s response to treatment. Since the KEYNOTE-522 subgroup analysis did not show a statistically significant difference in pCR for patients receiving carboplatin on an every 3 week schedule (difference in pCR 7.7%, 95% CI [− 5.0 to 20.6]) compared to those on a weekly dosing schedule (difference in pCR 18.4%, 95% CI [7.4 to 29.1]) [4], the utility of using carboplatin on an every 3 week schedule based on our results must be interpreted with caution. Additionally, it is important to note that the BrighTNness study, which examined the addition of carboplatin to neoadjuvant chemotherapy for TNBC, showed that carboplatin improves the pCR rate and event-free survival but not overall survival [21]. This likely explains why, as we show in this study of real-world practice patterns, carboplatin is the chemotherapy agent most commonly dropped from the KEYNOTE-522 regimen, as clinicians are trying to balance treatment-related toxicities while maximizing the long-term therapeutic benefit of treatment.

Overall, total rates of AEs in this study were lower than those reported in KEYNOTE-522 (88% compared to 99.5% in the study population), and the rate of grade 3+ AEs was significantly lower (38% compared to 81%) [4]. This is because a major limitation of this study is its retrospective nature. Since data were obtained from chart review, there is potential under-reporting of AEs, AE grade, steroid use, and hospitalizations, especially if a hospitalization was at an outside institution that did not share records with a patient’s treating physician. Additionally, it was sometimes difficult to determine if certain AEs were attributed to immunotherapy or to chemotherapy, so we reported rates of suspected irAEs and suspected multifactorial AEs. This notably included a total of 253 patients, or 61% of the total study population, which is a higher percentage than what was reported for irAEs in KEYNOTE-522 (33.5% for any grade irAE) [5]. The lack of differences in AEs or grade 3+ AEs based on race, age, or BMI are consistent with prior studies [14].

The majority of the AEs reported in the KEYNOTE-522 study occurred in the neoadjuvant treatment period [4], and in our study, the majority of the suspected irAEs or suspected multifactorial AEs (85%) also occurred in the neoadjuvant setting. It is likely, however, that the rate of suspected irAEs reported here is lower than what this patient population will eventually experience, as many patients in this cohort had either not started or were in the midst of adjuvant pembrolizumab treatment, and we collected data on irAEs experienced at any time after treatment initiation. As many irAEs from pembrolizumab, including endocrinopathies and nephritis, can occur late in the course of treatment [22], our study likely underestimates these rates due to the short length of follow up for many patients. Of the suspected irAEs or multifactorial AEs reported in the adjuvant setting in this study (50 reported events), the majority (32%) were endocrinopathies, although there were 12 reports of diarrhea/colitis (24%) and 4 cases (8%) of pneumonitis.

It is also important to acknowledge that 18 patients in this study died during our period of analysis. Sixteen of these deaths were due to progressive/metastatic breast cancer. These values may also be somewhat underrepresented due to the limited time frame of follow up in our study, as we collected patient information from 2021 to 2024, and many had not completed adjuvant therapy. Although it is impossible to statistically analyze this small number of patients, only one patient in this group obtained a pCR, and all others had residual disease. This finding highlights the aggressive nature of TNBC and the need for ongoing research into optimizing treatment regimens for TNBC patients, especially those who do not obtain a pCR following neoadjuvant treatment.

This analysis represents a diverse TNBC patient cohort who underwent treatment with neoadjuvant pembrolizumab and chemotherapy at seven academic medical centers in the United States. Our data highlight the high rates of complications and hospitalizations with this treatment approach as well as the difficulty with completing all planned doses of neoadjuvant chemotherapy and pembrolizumab and with adjuvant pembrolizumab, factors which likely contributed to the lower rate of pCR than those reported in other studies. This is also the first study to show that obese patients with TNBC have higher hospitalization rates when treated with combination chemotherapy and pembrolizumab. Ongoing research is needed to both determine which TNBC patients would specifically benefit from neoadjuvant combination chemotherapy and pembrolizumab in order to balance the risks and benefits of treatment and to fully understand the potential long-term side effects of immunotherapy in TNBC patients.

## Supplementary Information

Below is the link to the electronic supplementary material.

Supplementary file1 (DOCX 48 KB)

## Data Availability

The data generated and/or analyzed in this study are available from the corresponding author upon reasonable request.
