# Supplementary material for: Adverse events in patients treated with neoadjuvant chemo/immunotherapy for triple negative breast cancer: results from seven academic medical centers
Source: Breast Cancer Res Treat. 2025 Jul 4;213(1):71–80. doi: 10.1007/s10549-025-07758-8 (PMC12259778; doi:10.1007/s10549-025-07758-8)
Supplement: Supplementary file 1 — Supplementary file1 (DOCX 48 KB) [file 10549_2025_7758_MOESM1_ESM.docx]

**Adverse events in patients treated with neoadjuvant chemo/immunotherapy for triple negative breast cancer: results from seven academic medical centers**

Jessica Mezzanotte-Sharpe, MD, PhD^1^, Chih-Yuan Hsu, PhD^2^, David Choi, MD^3^, Hollie Sheffield, MD^4^, Sara Zelinskas, BS^5^, Ekaterina Proskuriakova, MD^6,7^, Mateo Montalvo, MD^8^, Danelle S. Lee, BA^8^, Jennifer G. Whisenant, PhD^1^, Keaton Gaffney, PharmD^9^, M. Scott Thompson, DPh^9^, Kim Blenman, PhD^8^, Karine Tawagi, MD^6,10^, Lynn Symonds, MD^11,12^, Cesar Santa-Maria, MD^3^, Nisha Unni, MD^4^, Dionisia Quiroga, DO, PhD^5^, Yu Shyr, PhD^2^, Laura C. Kennedy, MD, PhD^1^

Corresponding author:

Laura Kennedy, MD, PhD

Division of Hematology and Oncology, Vanderbilt University Medical Center

E-mail: [laura.kennedy@vumc.org](mailto:laura.kennedy@vumc.org)

**Supplemental Table 1: Surgical information**

SLNB: sentinel lymph node biopsy. ALND: axillary lymph node dissection
